# Coordinating ERK signaling via the molecular scaffold Kinase Suppressor of Ras

**DOI:** 10.12688/f1000research.11895.1

**Published:** 2017-08-31

**Authors:** Danielle Frodyma, Beth Neilsen, Diane Costanzo-Garvey, Kurt Fisher, Robert Lewis

**Affiliations:** 1 Eppley Institute for Research in Cancer and Allied Diseases, University of Nebraska Medical Center, Omaha, Nebraska, USA; 2 Department of Pathology and Microbiology, University of Nebraska Medical Center, Omaha, Nebraska, USA; 3 Fred & Pamela Buffett Cancer Center, University of Nebraska Medical Center, Omaha, Nebraska, USA

**Keywords:** ERK Signalling, Kinase Suppressor of Ras, KSR

## Abstract

Many cancers, including those of the colon, lung, and pancreas, depend upon the signaling pathways induced by mutated and constitutively active Ras. The molecular scaffolds Kinase Suppressor of Ras 1 and 2 (KSR1 and KSR2) play potent roles in promoting Ras-mediated signaling through the Raf/MEK/ERK kinase cascade. Here we summarize the canonical role of KSR in cells, including its central role as a scaffold protein for the Raf/MEK/ERK kinase cascade, its regulation of various cellular pathways mediated through different binding partners, and the phenotypic consequences of KSR1 or KSR2 genetic inactivation. Mammalian KSR proteins have a demonstrated role in cellular and organismal energy balance with implications for cancer and obesity. Targeting KSR1 in cancer using small molecule inhibitors has potential for therapy with reduced toxicity to the patient. RNAi and small molecule screens using KSR1 as a reference standard have the potential to expose and target vulnerabilities in cancer. Interestingly, although KSR1 and KSR2 are similar in structure, KSR2 has a distinct physiological role in regulating energy balance. Although KSR proteins have been studied for two decades, additional analysis is required to elucidate both the regulation of these molecular scaffolds and their potent effect on the spatial and temporal control of ERK activation in health and disease.

## Introduction

The three Ras genes ( *K-Ras*, *H-Ras*, and *N-Ras*) regulate diverse cellular functions through multiple pathways and are also commonly mutated in human cancer to yield constitutively active small GTPases. Activating Ras ** mutations are found in approximately 25% of human tumors, though these three small GTPases are not mutated at equivalent frequencies in cancer. A total of 85% of Ras-driven cancers have activating mutations in *K-Ras*, while *N-Ras* and *H-Ras* are mutated in 12% and 3%, respectively, of these cancers ( http://cancer.sanger.ac.uk/cosmic). Ras mutations are most common in pancreatic ductal adenocarcinomas (95%), colorectal adenocarcinomas (52%), and lung adenocarcinomas (31%). Intensive analysis revealed that multiple effectors with Ras-binding domains (or Ras association domains) were capable of interacting with the Ras effector loop and mediating its biological effects ^1^. Observations that activating Ras and Raf mutations are typically mutually exclusive ^2– 4^, and that only components of the Raf/MEK/ERK pathway rescue growth in “Rasless” mouse embryo fibroblasts (MEFs) ^5^, suggest that the interaction of Ras with Raf, and the activation of MEK1/2 and ERK1/2, may be most critical to Ras-driven cancers.

Kinase Suppressor of Ras 1 (KSR1) interacts with Raf, MEK, and ERK ^6– 12^, mediates ERK activation and signaling in a dose-dependent fashion (discussed in greater detail below), and is essential for the transformation of MEFs by oncogenic Ras ^8, 13^. These discoveries revealed a critical role played by this molecular scaffold in transformation and tumorigenesis. However, KSR1 ^–/–^ mice are fertile and show inconsequential developmental alterations ^12, 14^. These observations suggest that KSR1 may play a prominent role in cancers that are dependent upon Ras and ERK signaling and that it might be exploited therapeutically with minimal toxicity to the patient. Here we review the biochemistry and biology of KSR1 and its paralog, KSR2, and discuss their potential as therapeutic targets.

## The role of KSR proteins in the Raf/MEK/ERK cascade

A single *ksr* gene was identified as necessary for the rough-eye phenotype of activated Ras in *Drosophila* ^15^. Two *ksr* genes ( *ksr1* and *ksr2)* are expressed in *Caenorhabditis elegans* ^16, 17^ and mammals ^8, 15, 17– 20^. KSR1 and KSR2 proteins facilitate Raf phosphorylation of MEK, leading to increased ERK activation in response to Ras activation or calcium influx ^8, 9, 12, 15, 21– 25^. KSR proteins have properties expected of molecular scaffolds ^6– 12^. As expected of true scaffolds, increasing KSR1 allows for increased ERK activation until KSR1 reaches an optimal level. Surprisingly, in MEFs the level of KSR1 that maximizes ERK activation and signaling is approximately 12 times the endogenous level of expression. Further increasing KSR1 causes a decrease in ERK activation because the cellular concentration of KSR1 exceeds the amount of scaffold that can coordinate signaling with Raf, MEK, and ERK ^8, 26^. This suggests that overexpression of KSR1 sequesters individual components of the MAPK cascade such that they are unable to interact, which reduces MAPK signaling. However, overexpressing additional individual components of the MAPK pathway can suppress the inhibitory effects of scaffold excess ^9^. This observation likely explains why early studies in which ectopic KSR1 was overexpressed suggested that KSR1 inhibited Ras-driven transformation ^27– 30^. Importantly, the level of KSR1 expression that optimizes ERK activation is the same level that maximizes the transforming activity of oncogenic Ras and the proliferative effects of growth factors ^8^.

**Figure 1.**
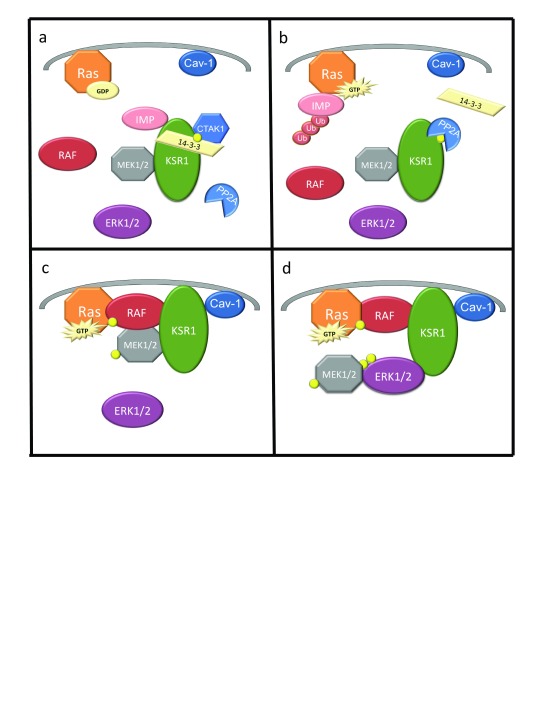
KSR1 dynamically regulates the Raf/MEK/ERK kinase cascade. ( **a**) KSR1 is constitutively bound to MEK1/2 and IMP. C-TAK1 phosphorylates (yellow circle) KSR1 at Ser392, allowing for 14-3-3 binding and cytoplasmic localization of KSR1. ( **b**) Upon Ras activation and GTP binding, IMP dissociates from KSR1, binds Ras, autoubiquitylates, and is degraded. PP2A dephosphorylates KSR1 at Ser392, destroying the 14-3-3 binding site anchoring KSR1. ( **c**) KSR1 and MEK1/2 translocate to the plasma membrane, where KSR1 interacts with Raf and MEK1/2 is phosphorylated and activated. ( **d**) MEK1/2 dissociates from KSR1 and ERK1/2 is phosphorylated and associates with KSR1, facilitating signaling. Cav-1, caveolin-1; C-TAK1, Cdc25C-associated kinase 1; ERK, extracellular signal-regulated protein kinase; GTP, guanosine triphosphate; IMP, impedes mitogenic signal propagation; KSR1, Kinase Suppressor of Ras 1; MEK, mitogen-activated protein/extracellular signal-regulated protein kinase kinase; PP2A, protein phosphatase 2.

Phosphorylation sites and determinants of protein–protein interaction have been mapped extensively on KSR1 and KSR2 and have been shown to regulate KSR1 in part through subcellular localization ^31– 37^. Analysis of these phosphorylation sites and interactions with effectors and modifiers suggest dynamic regulation of KSR1 and its scaffold function. Interaction with the E3 ligase IMP promotes the redistribution of KSR1 to Triton-resistant punctate structures that sequester KSR1 and impair ERK activation ^38, 39^. Phosphorylation of KSR1 on Ser297 and Ser392 (Ser310 and Ser469 in KSR2) by the kinase C-TAK1 (MARK3) creates a 14-3-3 binding site that anchors KSR1 within this subcellular compartment ( Figure 1a) ^13, 18, 25, 28, 38, 40– 42^. Ras activation catalyzes IMP autopolyubiquitylation and proteasomal destruction ( Figure 1b) ^39^. Stimuli that promote IMP degradation also promote the dephosphorylation of KSR1 at Ser392 by PP2A, which eliminates 14-3-3 binding ( Figure 1b) ^35, 41, 43^. Calcineurin dephosphorylates 14-3-3 binding sites on KSR2 ^25^. These events promote the redistribution of KSR1 to the plasma membrane, facilitating the activation of MEK by Raf ( Figure 1c). MEK is bound to KSR1 in the absence of Ras activation ^44^. Though identified as a loss-of-function mutation on KSR1 in *C. elegans* ^15, 16, 42^, the mutation of KSR1 at Cys809 to tyrosine (C809Y) enhances the activation of ERK in mammalian cells ^13^. These observations suggest that KSR proteins may sequester MEK in an inactivated state and present MEK for phosphorylation by Raf ^21, 45^. In this model, MEK does not need to be in complex with KSR1 to phosphorylate and activate ERK. However, KSR1 encodes a DEF domain ^46^, which is essential to KSR1-mediated ERK interaction and critical for competent signal transduction ^13, 34^. KSR1 (but not KSR2) also encodes a caveolin-binding domain ^47^, which is required for binding to caveolin-1, localization to the plasma membrane, and ERK activation ( Figure 1c and 1d) ^47, 48^. Another level of KSR1 regulation exists in its degradation. Recently, it has been shown that KSR1 is polyubiquitinated by praja2, which promotes KSR1 degradation, causing a decrease in ERK signaling ^49^. Reconciling these observations implies that KSR1 coordinates a dynamic mechanism that provides spatial and temporal control of signaling through the Raf/MEK/ERK kinase cascade.

## Regulation of MEK and ERK by KSR and Raf heterodimers

The dimerization of Raf proteins is an essential step in wild-type Raf activation. KSR and Raf proteins share homology within the region required for Raf dimerization, and KSR has been shown to form heterodimers with Raf, particularly B-Raf ^24, 36, 50^. Based on modeling, KSR dimerization with Raf induces a conformational change in KSR that induces the exposure of the MEK activation loop and facilitates its phosphorylation ^24^. However, the dimerization of KSR and Raf orients the Raf protein such that the catalytic site of Raf is not in close proximity to its phosphorylation target site on MEK. Therefore, MEK phosphorylation must be completed by another Raf protein ^24, 36^. KSR2 also forms homodimers through a side-to-side interface that is specifically dependent upon Arg718 ^36^. Mutations at this site suppress Ras signaling, suggesting that the dimerization of KSR proteins is required to promote Ras signaling ^24^. This observation is consistent with results showing that mutations inhibiting the KSR-Raf heterodimerization decrease Raf activity ^36^. Additionally, there is potential for KSR1 to directly activate ERK or BRAF and CRAF if Y552 is phosphorylated, as the KSR1 ^Y552D^ mutant demonstrated this ability ^51^. The functional role of KSR homodimers is still incompletely understood, but the ability of IMP to inhibit MEK activation by Raf has been suggested to result from IMP-mediated disruption of KSR1 homodimers and B-Raf/c-Raf heterodimers ^52^.

## Phenotypic effects of KSR1/2 genetic inactivation

Genetic studies in model organisms demonstrate that KSR proteins promote Ras signaling ^15– 17^. Heterozygous loss of *ksr* in *Drosophila* suppresses Ras ^G12V^ signaling and prevents the rough-eye phenotype caused by constitutive Ras signaling ^15, 28^. Similarly, loss-of-function mutations in *ksr1* suppressed the multiple vulva phenotype of activated Ras in *C. elegans* ^43^. KSR1 plays a similar role in mammals. Apart from minor deficits, *ksr1* ^–/–^ knockout mice are fertile and developmentally normal. *Ksr1 ^–/–^* mice have hair follicle defects similar to the phenotype of *egfr* ^–/–^ mice, supporting the suggestion that these proteins function within the same pathway ^12, 14, 53^. As a result of reduced ERK signaling, *ksr1* ^–/–^ mice have a marginally impaired immunological response ^6, 12, 54, 55^. The most profound and translationally significant phenotype of *ksr1* ^–/–^ mice is resistance to Ras-dependent tumor formation. Skin tumor induction by v-Ha-Ras is lost in *ksr1* ^–/–^ mice ^14^, and mammary tumor burden is markedly reduced by KSR1 disruption in mice expressing transgenic polyomavirus Middle T-Antigen ^12^. These observations demonstrate that KSR modulates Ras signaling *in vivo*, but it is largely dispensable for normal cell survival. The requirement for KSR1 in Ras-driven tumor formation, but not normal development, reveals KSR1 as a potential target for therapeutic intervention.

In contrast to the mild phenotype of *ksr1* ^–/–^ mice, *ksr2 ^–/–^* mice have reduced fertility and become spontaneously obese ^56– 59^. Pathways regulating adaptive thermogenesis, metabolic rate, and leptin-sensitive food consumption are implicated in KSR2-dependent energy balance ^56– 60^. Consistent with observations in the knockout mice, humans with *ksr2* mutations show severe early onset obesity ^60^. *Ksr2* variants in humans that impair Ras signaling or inhibit KSR2 interaction with AMPK also disrupt glucose metabolism and fatty acid oxidation ^60^. Interestingly, KSR2 is almost exclusively expressed in the brain and pituitary ^19, 58^. Brain-specific disruption of KSR2 is sufficient to cause obesity and glucose intolerance in mice, though it does not perfectly recapitulate the phenotype of *ksr2 ^–/–^* mice ^19^. These observations show that KSR2 function in the brain plays a potent role in the regulation of energy balance.

## Structural properties of KSR proteins

KSR1 and KSR2 proteins are highly conserved in invertebrates and mammals ^9, 15^. KSR proteins are structurally related to Raf proteins in five conserved areas, CA1–CA5 ^15^. CA1 is located on the N-terminus end. It contains 40 amino acids that contribute to B-Raf binding by KSR1 and encode coiled-coil and sterile-α-motif (SAM) structures that promote KSR1 membrane association ^13, 34, 44, 61^. CA2 is a proline-rich region without known function. A region in KSR2 between CA2 and CA3 is required for KSR interaction with AMPK, and mutations in this region inhibit this interaction ^19, 31, 58^. CA3 is a cysteine-rich region containing an atypical C1 motif homologous to the cysteine-rich CR1 region in Raf that also contributes to KSR1 membrane localization ^21, 62^. CA3 mediates the membrane localization of KSR by recruiting phospholipids but does not react to phorbol esters or ceramide or interact directly with Ras ^63^. CA4 is a serine/threonine-rich region that mediates interactions with ERK through an FXFP motif ^32^. This interaction is not constitutive but requires Ras activation ^21, 33, 42, 61, 63^. The CA5 domain in KSR proteins encodes a kinase (or pseudokinase) domain highly homologous to Raf family CR3 kinase domains ^15, 17^. Mammalian KSR proteins contain multiple alterations in amino acids typically required for catalytic activity including arginine in place of the lysine that coordinates the gamma phosphate of ATP ^15, 61^. Substantial effort has been exerted to clarify if KSR can or does phosphorylate cellular substrates and, if so, whether or not this activity contributes to the downstream effects of KSR ^21, 24, 37, 64^. KSR1 substrates and the biological relevance of any residual phosphotransferase activity have yet to be validated.

The CA5 region contributes to KSR interaction with MEK in both quiescent and growth factor-activated cells ^31, 44, 65^. Amino acid substitutions within the CA5 region that diminish interaction with MEK also reduce ERK signaling ^15– 17, 44, 65^. However, these alterations are within or near the ATP-binding domain and may disrupt ATP binding, potentially affecting interaction with MEK secondarily. The CA5 domain also interacts with Raf, but the mechanism is incompletely understood ^34^. Thus, there may be unidentified dynamic interactions between the CA1 and CA5 domains of KSR proteins and B-Raf that regulate Raf kinase activation, MEK phosphorylation, and signal transduction through the kinase cascade.

## KSR proteins as targets for therapy

Given the importance of KSR1 in modulating signaling through the Raf/MEK/ERK kinase cascade in tumor cells and observations that *ksr1 ^–/–^* mice develop with only inconsequential phenotypic differences, targeting KSR1 or KSR1-dependent signaling pathways in Ras-driven cancers may selectively target cancer cells with reduced toxicity to patients. Supporting this strategy, RNAi approaches depleting cancer cells of KSR1 *in vitro* and *in vivo* caused a decrease in tumor growth. Continuous infusion of phosphorothioate antisense oligonucleotides targeting KSR1 mRNA also caused regression of established tumors and inhibited metastases without overt toxicity in Ras-driven PANC-1 pancreatic and A549 non-small-cell lung cancer xenografts ^66^.

Mutations in KSR that suppress signaling by activated Ras are often adjacent to the ATP-binding pocket ^15– 17^. Furthermore, KSR1 binds ATP ^65^, and mutations that prevent that binding impair ERK activation ^67^. These observations suggest that manipulation of the ATP-binding cleft in KSR1 may be therapeutically effective. The recently discovered small molecule APS-2-79 binds and stabilizes KSR kinase domains in an inactive conformation observed when the KSR2 kinase domain is bound to MEK1 and ATP ^24^, interferes with KSR:Raf heterodimerization, and inhibits oncogenic Ras signaling ^50^. The effect of APS-2-79 was also lost when KSR was mutated within the active site (A690F) such that KSR can promote MEK phosphorylation even in the absence of ATP binding. APS-2-79 modestly decreased cell viability in two Ras-mutated cancer cell lines (HCT116 and A549) and did not affect Raf-mutated cancer cells (A375 and SK-MEL-293) but did demonstrate substantial synergy in Ras-mutated cancer cells with MEK inhibitors, suggesting the potential to target both kinase and scaffolding components of the Ras signaling pathway in Ras-dependent cancers ^50^.

The observation that KSR1 expression was required for tumor-dependent ERK signaling, but not normal development, suggested the possibility that effectors of KSR1-dependent signaling pathways in tumor cells might reveal additional putative targets for cancer therapy that preferentially support tumor cell growth and viability. A gene expression high-throughput screen termed Functional Signature Ontology (FUSION) was developed to detect effectors of KSR1-dependent signaling in Ras-driven tumors and identify small molecules that can target those effectors ^68^. Recent results from FUSION detected hits that mediate KSR1-dependent signals and promote the viability of human colon tumor cells but have no similar role on non-transformed human colon epithelial cells ^69, 70^. These observations suggest the existence of multiple effectors that may be used to support the survival in tumor cells in a manner distinct from their role in normal tissue. Careful validation of these potential targets may yield additional therapeutic strategies.

## Conclusions

KSR proteins are established as scaffolds for the Raf/MEK/ERK kinase cascade, though a detailed understanding of KSR-dependent temporal and spatial control of ERK signaling is still lacking. The physiology regulated by KSR1 and KSR2 is also incompletely understood, though disruption of KSR2 in mice and its mutation in humans show that it plays an important role in mammalian energy balance. Given the minimal phenotype of *ksr1 ^–/–^* mice, the normal function of KSR1 is unclear. However, KSR1 is demonstrably physiologically important as *ksr1 ^–/–^ ksr2 ^–/–^* mice do not survive beyond 21 days of age (Costanzo-Garvey and Lewis, unpublished results), while the single gene knockouts survive into adulthood. KSR1 and KSR2 are primarily expressed in brain, though KSR1 is expressed at lower levels throughout the body. Thus, the most profound function of KSR proteins is likely found in the central nervous system. The obesity phenotype caused by brain-specific knockout of KSR2 demonstrates its potent role in cell non-autonomous regulation of energy balance. However, the role of KSR2 as a scaffold and its regulation of ERK signaling in support of brain-regulated development and maintenance of metabolism are unknown. Relevant *in vitro* cell models that express both KSR1 and KSR2 will be crucial to further our understanding of the cell biology controlled by these molecular scaffolds.
